# Social and Financial Costs of Neonatal Intestinal Failure

**DOI:** 10.1001/jamanetworkopen.2024.59548

**Published:** 2025-02-26

**Authors:** Vikram K. Raghu, Sirine Belaid, Susan Gutierrez, Pamela Holzer, Shelby Orris, Scott Rothenberger, Tracey Presel, Kimberly Ackerman, Feras Alissa, Dale King, Jennifer Woo Baidal, Jeffrey A. Rudolph, Geoffrey Bond, George V. Mazariegos, Simon P. Horslen, Kenneth J. Smith

**Affiliations:** 1 Department of Pediatrics, University of Pittsburgh School of Medicine, Pittsburgh, Pennsylvania; 2 Division of Pediatric Gastroenterology, UPMC Children’s Hospital of Pittsburgh, Pittsburgh, Pennsylvania; 3 Department of Pediatrics, University of California, San Francisco, San Francisco; 4 Department of Medicine, University of Pittsburgh School of Medicine, Pittsburgh, Pennsylvania; 5 Department of Pediatrics, Stanford University, Palo Alto, California; 6 Department of Surgery, University of Pittsburgh School of Medicine, Pittsburgh, Pennsylvania; 7 Division of Pediatric Transplantation, UPMC Children’s Hospital of Pittsburgh, Pittsburgh, Pennsylvania

## Abstract

**Importance:**

Neonatal short bowel syndrome with intestinal failure is rare, and observational studies are limited to small cohorts. Health disparities exist in long-term management with little known about the initial hospitalization.

**Objective:**

To identify children with neonatal short bowel syndrome with intestinal failure and to determine whether race, ethnicity, and neighborhood opportunity are associated with length of stay during their initial hospitalizations.

**Design, Setting, and Participants:**

This cross-sectional study of children with neonatal short bowel syndrome and intestinal failure was conducted between 2004 and 2020, with validation and analysis performed from July 2022 to April 2024. Children were identified from the Pediatric Health Information System database, which included administrative data from 50 freestanding US children’s hospitals. Children were included on the basis of a diagnosis code of postsurgical malabsorption and billed charges for a neonatal intensive care unit stay and sustained parenteral nutrition use.

**Exposures:**

Race and ethnicity were included as a single variable with categories of Hispanic, non-Hispanic Black, non-Hispanic White, other (Asian and multiracial, combined because of low numbers), and unknown according to hospital report. Child Opportunity Index quintile ranged from very low (lowest quintile) to very high (highest quintile).

**Main Outcomes and Measures:**

The primary outcome was length of stay measured in number of days from admission to discharge. The χ^2^ analysis was used to examine unadjusted associations between categorical variables, and Wilcoxon rank-sum test was used for continuous variables.

**Results:**

A total of 2267 children with neonatal short bowel syndrome with intestinal failure were identified (997 female [44%]; 410 Hispanic [18%]; 481 non-Hispanic Black [21%]; 690 non-Hispanic White [30%]; 231 other [10%]; 455 unknown [20%]), with 629 (28%) living in areas with very low Child Opportunity Index. The median (IQR) length of stay for the initial admission was 150 (112-200) days, with a median (IQR) cost of $528 628 ($374 040-$766 446). In multivariable analysis, non-Hispanic Black children remained in the hospital for 16 days longer than their non-Hispanic White counterparts (95% CI, 7-25 days; *P* < .001). Child Opportunity Index was not significantly associated with length of stay when controlling for race and ethnicity.

**Conclusions and Relevance:**

In this cross-sectional study, children with neonatal short bowel syndrome with intestinal failure experienced long initial hospital stays, incurring high costs, with non-Hispanic Black children disproportionately affected. Interventions targeting safe and efficient transition to home are needed to address both efficacy and equity for these children.

## Key Points

**Question:**

What characteristics are associated with prolonged initial length of stay for children with neonatal short bowel syndrome with intestinal failure?

**Findings:**

This cross-sectional analysis using a novel algorithm to identify 2267 children with short bowel syndrome with intestinal failure found that non-Hispanic Black children were hospitalized 16 days longer than non-Hispanic White children, independently of diagnosis and complications.

**Meaning:**

These findings suggest that children with intestinal failure experience race-based disparities in care that require intervention.

## Teaser

This cross-sectional study examines whether race, ethnicity, and neighborhood opportunity are associated with length of stay during their initial hospitalizations among children with neonatal short bowel syndrome with intestinal failure.

## Introduction

Children with intestinal failure due to neonatal short bowel syndrome receive stark variations in care, with little known about the reasons.^1^ Intestinal failure is a heterogenous condition in which an individual cannot meet the minimal nutrition needs for growth and development, thus requiring parenteral nutrition.^2,3^ The most common cause of pediatric intestinal failure is neonatal short bowel syndrome, where a child has decreased intestinal length for congenital or surgical reasons.^1,4,5^ Studying children with intestinal failure from short bowel syndrome is challenging owing to a lack of a precise diagnosis or definition. Existing cohort studies include retrospective cohorts of up to 400 children and prospective cohorts of approximately 200 children.^1,4,5,6^ Recently, the American Society for Parenteral and Enteral Nutrition published consensus definitions around short bowel syndrome and intestinal failure based on cause of short bowel syndrome and duration of parenteral nutrition.^3^ The *International Statistical Classification of Diseases and Related Health Problems, Tenth Revision (ICD-10)* recently incorporated these definitions into new diagnosis codes for short bowel syndrome and intestinal failure. However, data contained in large administrative databases lack specificity to identify these patients.

The Pediatric Health Information Systems (PHIS) database presents one such opportunity to study neonatal short bowel syndrome. Uniquely, PHIS contains daily charges associated with hospital encounters from approximately 50 freestanding children’s hospitals. With these data, a combination of diagnosis and parenteral nutrition use may be used to identify neonates with short bowel syndrome.

In the absence of data describing practice patterns in short bowel syndrome, great variability in care occurs, at times leading to health disparities. In children studied from 2000 to 2006, Black children had greater mortality and less frequently underwent transplant than White children.^7^ More recently, central line–associated bloodstream infection (CLABSI), a common yet potentially life-threatening complication of short bowel syndrome, was found to occur more commonly among minoritized children and those with lower socioeconomic status.^8^

In this study, we use the newly published definitions of pediatric intestinal failure to identify a cohort with intestinal failure due to neonatal short bowel syndrome in the PHIS database. With this cohort, we examined the association between their social, demographic, and clinical characteristics with health care utilization during their initial hospital stay.

## Methods

We performed a cross-sectional analysis of the initial hospitalization for children with short bowel syndrome with intestinal failure from 2004 to 2020. This study was approved by the institutional review board of the University of Pittsburgh under exempt category 4 by 45 CFR §46. Consent was not obtained because the data were obtained from a deidentified source. This report follows the Strengthening the Reporting of Observational Studies in Epidemiology (STROBE) reporting guideline.^9^

We identified children in the PHIS database with a diagnosis code of postsurgical malabsorption, neonatal intensive care unit stay, and parenteral nutrition use for 60 days discharged between 2004 and 2020. In that time frame, postsurgical malabsorption, a diagnosis code previously used to identify patients with short bowel syndrome, included both the *International Classification of Diseases, Ninth Revision (ICD-9)* code 579.3 and the *ICD-10 *code K91.2.^10,11^ Neonatal intensive care unit stay was identified using a PHIS flag linked to a neonatal intensive care unit day billing code. Daily pharmacy charges identified patients who received parenteral nutrition for 60 days over a 74-day period before discharge as per the ASPEN definition.^3^ We developed an algorithm in R statistical software version 4.3.3 (R Project for Statistical Computing) to identify this cohort from all neonates with a diagnosis code of postsurgical malabsorption with any billing code for parenteral nutrition. The Figure shows the cohort selection procedure. Once identified using the algorithm, the subset from our hospital was validated by 2 clinical staff to confirm that diagnostic criteria were met for short bowel syndrome with intestinal failure.^3^

**Figure.**
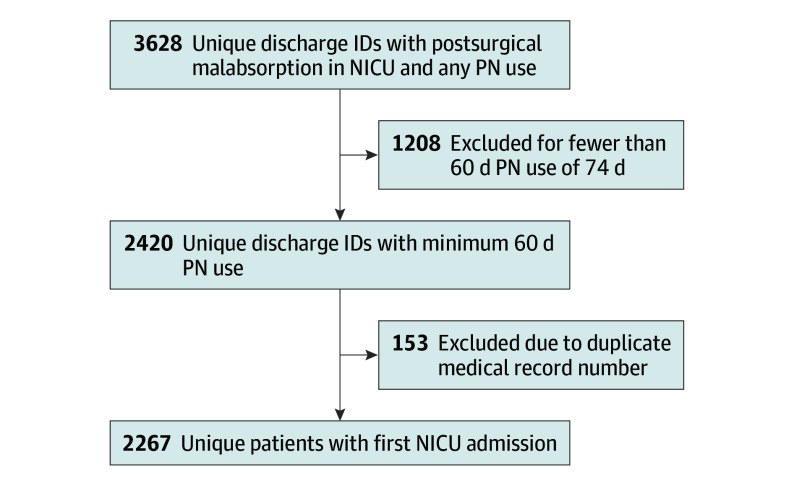
Study Flow Diagram ID indicates identification; NICU, neonatal intensive care unit; PN, parenteral nutrition.

Demographics, diagnosis and procedures codes, utilization, and Child Opportunity Index (COI) were collected. The COI combines 29 indicators (eg, school poverty, household income, and air pollution levels) to represent neighborhood opportunity categorized into 5 levels from very low to very high.^12^ Sex was categorized as male or female. The social constructs of race and ethnicity are reported in PHIS as a single categorical variable with categories of Asian, Hispanic, non-Hispanic Black, non-Hispanic White, multiracial, and unknown to follow the standard of the United Network for Organ Sharing. Asian and multiracial were combined into a single other category given the small numbers in each category. Payer was categorized into government, commercial, or other or unknown. Gestational age was recorded in weeks, with median imputation performed for those with missing values. We chose a dichotomous variable for era with 2010 as the demarcation, corresponding with mainstream use of fish oil–based lipid emulsions, which were associated with reductions in cholestasis and the need for urgent transplant.^13^ We identified 5 diagnosis categories for neonatal short bowel syndrome with intestinal failure: necrotizing enterocolitis, gastroschisis, atresia, Hirschsprung disease, and volvulus. We treated them as independent factors since patients may belong to multiple categories. Diagnoses were identified by *ICD-9* and *ICD-10* codes. We identified 2 common complications among neonates with intestinal failure—CLABSI and venous thromboembolism (VTE)—by diagnosis codes.

### Statistical Analysis

Validation and analysis were performed from July 2022 to April 2024. Descriptive statistics characterized all variables. We used χ^2^ analysis to examine unadjusted associations between categorical variables and the Wilcoxon rank-sum test for continuous variables. Our primary outcome was in-hospital length of stay in days during the primary admission. Secondary outcomes included in-hospital mortality and total standardized costs adjusted to 2021 US dollars. The standardized cost method applies a single median cost value across all hospitals to each charge, rather than using hospital-specific cost-to-charge ratios. We fit a mixed-effects generalized linear regression with γ family distribution and log link to examine factors associated with increased length of stay and increased costs. A random intercept term was used to account for center variations in practice. We fit a Cox proportional hazards model with censoring at discharge to determine factors associated with in-hospital mortality. In this model, we accounted for center variation through a cluster variance-covariance estimator that allowed for correlation between individuals at the same site. For all models, we used univariable selection with a threshold of *P* < .20 followed by backward selection using a likelihood ratio test with a threshold of *P* < .05 to arrive at the final model. Statistical analyses were performed in Stata statistical software version 18 (StataCorp). We performed 3 sensitivity analyses on our model selection. First, we used a least absolute shrinkage and selection operator in place of our backward variable selection. This was not performed as the primary variable selection method because we were unable to incorporate a γ distribution in the linear model selection and were unable to account for clustering in the variance-covariance estimator in the Cox model selection. Instead, we used a simple linear model with log-transformed length of stay to perform variable selection as a surrogate for the γ distribution generalized linear model. Second, we performed backward selection only including those with nonmissing gestational age prior to any imputation. Third, we performed backward selection only including those in the more recent era. Specifically for length of stay, a fourth sensitivity analysis was performed only for patients who survived the initial hospital stay to determine whether death as an informative censoring event influenced the model.

## Results

We identified 2267 neonates (997 female [44%]; 410 Hispanic [18%]; 481 non-Hispanic Black [21%]; 690 non-Hispanic White [30%]; 231 other [10%]; 455 unknown [20%]) with intestinal failure from short bowel syndrome. Of the 51 children in the cohort who came from UPMC Children’s Hospital of Pittsburgh, 50 were confirmed to meet diagnostic criteria for short bowel syndrome with intestinal failure with 1 whose data predated the electronic health record and could not be confirmed.

Table 1 contains the descriptive features of the full analysis cohort. Non-Hispanic Black children were overrepresented in this cohort compared with the general population. Very low COI included 28% of the cohort (629 children). Most children had government insurance. The cause of intestinal failure was most commonly necrotizing enterocolitis. In the subset with a reported gestational age, the median (IQR) gestational age was 31 (26-35) weeks. Those identified as non-Hispanic Black race had more frequent diagnosis of necrotizing enterocolitis (305 of 481 children [63%] vs 862 of 1786 children [48%]; χ^2^ = 34.80; *P* < .001), lower gestational age (median [IQR], 31 [23-31] weeks vs 31 [28-34] weeks), and low or very low COI (318 of 481 children [66%] vs 835 of 1786 children [47%]; χ^2^ = 56.80; *P* < .001) compared with children from the other racial and ethnic groups.

**Table 1.** Demographic and Clinical Characteristics of Children With Short Bowel Syndrome and Intestinal Failure

Characteristic	Children, No. (%) (N = 2267)
Sex	
Female	997 (44)
Male	1370 (56)
Gestational age, median (IQR), wk	31 (26-35)
No. for whom gestational age was known	1639
Race and ethnicity	
Hispanic	410 (18)
Non-Hispanic Black	481 (21)
Non-Hispanic White	690 (30)
Other^a^	231 (10)
Unknown	455 (20)
Child Opportunity Index	
Very low	629 (28)
Low	524 (23)
Moderate	448 (20)
High	377 (17)
Very high	270 (12)
Unknown	19 (1)
Payer	
Commercial	530 (23)
Government	1272 (56)
Other or unknown	465 (21)
Admission prior to 2010	747 (33)
Diagnosis	
Necrotizing enterocolitis	1167 (51)
Gastroschisis	307 (14)
Volvulus	104 (5)
Hirschsprung disease	35 (2)
Atresia	526 (23)
Complications	
Central line–associated bloodstream infection	304 (13)
Venous thromboembolism	216 (10)
Death	152 (7)
Length of stay, median (IQR), d	150 (112-200)
Cost, median (IQR), $	528 628 (374 040-766 446)

^a^ Other includes Asian, multiracial, and other categories from the original dataset.

For outcomes, 304 children (13%) had a CLABSI prior to their first discharge, and 216 (10%) had VTE. Non-Hispanic Black children had high rates of CLABSI (66 of 481 children [16%] vs 229 of 1786 children [13%]; χ^2^ = 13.47; *P* < .001) and VTE (66 of 481 children [14%] vs 150 of 1786 children [8%]; χ^2^ = 12.50; *P* < .001) compared with children from other racial and ethnic groups. Mortality was 7% (152 deaths) during the first hospital stay. Non-Hispanic White children had lower mortality than children from the other racial and ethnic groups (29 of 690 children [4%] vs 124 of 1577 children [8%]; χ^2^ = 29.60; *P* < .001). Children had a median (IQR) length of stay of 150 (112-200) days. The median (IQR) admission cost was $528 628 ($374 040-$766 446).

Table 2 shows factors associated with increased length of stay. In the final multivariable model race and ethnicity, COI, payer, gestational age, necrotizing enterocolitis diagnosis, CLABSI, and VTE were associated with increased length of stay. Specifically, non-Hispanic Black children were hospitalized 16 days longer (95% CI, 7-25 days; *P* < .001) than non-Hispanic White children when adjusting for other factors. Those with government insurance were hospitalized 10 days longer than those with commercial insurance. Those with necrotizing enterocolitis as the cause of intestinal failure were hospitalized 15 days longer than those with other causes. Those with unknown COI were hospitalized longer than other children, but there were no significant differences between known COI levels. Every additional week of gestation was associated with a 2-day reduction in length of stay. Finally, children with either a CLABSI or VTE were hospitalized 24 days longer than those without each complication. All 3 sensitivity analyses resulted in similar significant associations between length of stay and race, payer, diagnosis, CLABSI, and VTE, except that there were no significant associations between payer and length of stay when those with missing gestational age were excluded (eTable 1 in Supplement 1). Results of the fourth sensitivity analysis (omitting patients who died) had no effect on model selection. The final model estimates were of similar magnitude as the original model and thus not reported.

**Table 2.** Association Between Social and Clinical Variables and Initial Length of Stay

Variable	Univariable		Multivariable	
	Marginal length of stay, d (95% CI)	*P* value	Marginal length of stay, d (95% CI)	*P* value
Female sex (vs male sex)	3 (−3 to 8)	.43	NA	NA
Gestational age, wk	−3 (−4 to −2)	<.001	−2 (−3 to −2)	<.001
Race and ethnicity				
Hispanic	13 (4 to 22)	<.001	11 (1 to 20)	<.001
Non-Hispanic Black	27 (18 to 36)		16 (7 to 25)	
Non-Hispanic White	0 [Reference]		0 [Reference]	
Other^a^	11 (1 to 22)		7 (−3 to 17)	
Unknown	6 (−2 to 14)		−4 (−15 to 7)	
Child Opportunity Index				
Very low	0 [Reference]	<.001	0 [Reference]	.03
Low	−7 (−16 to 1)		−2 (−10 to 6)	
Moderate	−7 (−15 to 2)		1 (−7 to 10)	
High	−6 (−15 to 3)		3 (−6 to 12)	
Very high	−17 (−27 to −7)		−8 (−18 to 2)	
Unknown	59 (14 to 103)		54 (12 to 96)	
Payer				
Government	0 [Reference]	<.001	0 [Reference]	<.001
Commercial	−14 (−21 to −7)		−10 (−17 to −3)	
Other or unknown	0 (−8 to 8)		12 (0 to 23)	
Admission after 2009	−4 (−10 to 3)	.26	NA	NA
Diagnosis				
Necrotizing enterocolitis	23 (17 to 29)	<.001	15 (9 to 21)	<.001
Gastroschisis	−7 (−15 to 2)	.12	NA	NA
Volvulus	−14 (−27 to −1)	.04	NA	NA
Hirschsprung disease	4 (−20 to 28)	.74	NA	NA
Atresia	−13 (−19 to −6)	<.001	NA	NA
Complications				
Central line–associated bloodstream infection	27 (17 to 36)	<.001	24 (14 to 33)	<.001
Venous thromboembolism	29 (18 to 40)	<.001	24 (13 to 34)	<.001

Abbreviation: NA, not applicable.

^a^ Other includes Asian, multiracial, and other categories from the original dataset.

Table 3 depicts factors associated with increased costs. In the final model, each additional day in hospital length of stay was associated with a marginal cost of $3791 (95% CI, $3172-$4410). Other factors associated with cost independently of length of stay included COI, payer, gestational age, era, and diagnosis. Race and ethnicity and complications such as CLABSI and VTE were not associated with a difference in cost independently of length of stay. In sensitivity analyses, only length of stay and diagnosis had robust associations with total cost (eTable 2 in Supplement 1).

**Table 3.** Association Between Social and Clinical Variables and Standardized Total Cost

Variable	Univariable		Multivariable	
	Marginal cost, $ (95% CI)	*P* value	Marginal cost, $ (95% CI)	*P* value
Length of stay, d	3954 (3277 to 4630)	<.001	3791 (3172 to 4410)	<.001
Female sex (vs male sex)	−19 933 (−60 467 to 20 600)	.48	NA	NA
Gestational age, wk	−18 257 (−23 874 to −12 639)	<.001	−5823 (−10 083 to −1563)	.007
Race and ethnicity				
Hispanic	1923 (−62 829 to 66 675)	<.001	NA	NA
Non-Hispanic Black	76 835 (11 575 to 142 094)			
Non-Hispanic White	0 [Reference]			
Other^a^	−9460 (−84 009 to 65 089)			
Unknown	−158 186 (−216 255 to −100 117)			
Child Opportunity Index				
Very low	0 [Reference]	.17	0 [Reference]	.007
Low	3322 (−55 115 to 61 760)		22 493 (−22 730 to 67 717)	
Moderate	−55 580 (−115 546 to 4387)		−28 997 (−75 508 to 17 515)	
High	16 071 (−48 760 to 80 902)		38 830 (−12 824 to 90 484)	
Very high	13 703 (−59 716 to 87 121)		82 970 (21 181 to 144 759)	
Unknown	148 223 (−123 795 to 420 241)		11 411 (−165 943 to 188 766)	
Payer				
Government	0 [Reference]	<.001	0 [Reference]	<.001
Commercial	−101 597 (−152 869 to −50 324)		−55 069 (−96 763 to −13 375)	
Other or unknown	−198 818 (−254 640 to −142 996)		−107 335 (−160 835 to −53 835)	
Admission after 2009	177 641 (129 178 to 226 104)	<.001	136 798 (98 158 to 194 264)	<.001
Diagnosis				
Necrotizing enterocolitis	125 161 (79 098 to 171 224)	<.001	42 168 (6721 to 77 615)	.02
Gastroschisis	70 388 (4032 to 136 745)	.03	78 495 (20 915 to 136 074)	.004
Volvulus	−170 919 (−251 942 to −89 896)	<.001	−114 275 (−181 925 to −46 625)	.002
Hirschsprung disease	8082 (−157 152 to 173 316)	.92	NA	NA
Atresia	−46 102 (−94 556 to 2352)	.06	NA	NA
Complications				
Central line–associated bloodstream infection	91 718 (24 089 to 159 347	.004	NA	NA
Venous thromboembolism	199 106 (109 254 to 288 958)	<.001	NA	NA

Abbreviation: NA, not applicable.

^a^ Other includes Asian, multiracial, and other categories from the original dataset.

Table 4 shows Cox proportional hazards model results examining associations with in-hospital mortality. In the final model, race and ethnicity, gastroschisis and atresia diagnoses, CLABSI, and VTE were significantly associated with mortality. Children with unknown race and ethnicity had 2.35 times the hospital mortality risk of those identified as non-Hispanic White. Those with VTE had 1.80 times the hospital mortality risk. Children with CLABSI had a lower mortality risk than those without. Children with atresia and gastroschisis were 2 and 3 times more likely to survive their initial hospital stay, respectively, compared with children without these diagnoses. In sensitivity analyses, a consistent association was observed between in-hospital mortality risk and VTE (eTable 3 in Supplement 1). In the model with only those with nonmissing gestational age, later gestational age was associated with lower mortality risk.

**Table 4.** Association Between Social and Clinical Variables and Mortality

Variable	Univariable		Multivariable	
	HR (95% CI)	*P* value	HR (95% CI)	*P* value
Female sex (vs male sex)	0.98 (0.71-1.34)	.88	NA	NA
Gestational age, wk	0.97 (0.95-0.99)	.01	NA	NA
Race and ethnicity				
Hispanic	1.26 (0.64-2.50)	<.001	1.30 (0.64-2.62)	<.001
Non-Hispanic Black	1.26 (0.63-2.53)		1.07 (0.53-2.15)	
Non-Hispanic White	1 [Reference]		1 [Reference]	
Other^a^	1.09 (0.48-2.48)		1.12 (0.50-2.48)	
Unknown	2.65 (1.47-4.78)		2.35 (1.30-4.27)	
Child Opportunity Index				
Very low	1 [Reference]	.17	NA	NA
Low	0.93 (0.62-1.38)			
Moderate	0.69 (0.37-1.25)			
High	0.53 (0.32-0.87)			
Very high	0.75 (0.41-1.39)			
Unknown	0.79 (0.31-2.04)			
Payer				
Government	1 [Reference]	<.001	NA	NA
Commercial	1.39 (0.89-2.16)			
Other or unknown	2.48 (1.66-3.69)			
Admission after 2009	0.50 (0.33-0.76)	.001	NA	NA
Diagnosis				
Necrotizing enterocolitis	1.08 (0.77-1.50)	.66	NA	NA
Gastroschisis	0.24 (0.10-0.55)	<.001	0.37 (0.17-0.80)	.01
Volvulus	0.92 (0.33-2.55)	.87	NA	NA
Hirschsprung disease	0.70 (0.17-2.96)	.63	NA	NA
Atresia	0.46 (0.28-0.76)	.002	0.57 (0.35-0.91)	.02
Complications				
Central line–associated bloodstream infection	0.54 (0.38-0.77)	<.001	0.58 (0.39-0.85)	.006
Venous thromboembolism	1.69 (1.12-2.56)	.01	1.80 (1.22-2.63)	.003

Abbreviations: HR, hazard ratio; NA, not applicable.

^a^ Other includes Asian, multiracial, and other categories from the original dataset.

## Discussion

Children with neonatal short bowel syndrome resulting in intestinal failure require intense resource utilization during their initial hospitalization. In this first cross-sectional report utilizing the PHIS database and adhering to the new consensus definition of intestinal failure defined by the American Society of Parenteral and Enteral Nutrition, we validated a novel approach to successfully identify children with intestinal failure according to in-hospital parenteral nutrition use. This cohort of more than 2200 children across 50 hospitals is much larger than previous cohorts and can be used for longitudinal study of hospital-related outcomes. These children required hospital stays that lasted 5 months and cost more than $500 000. They had high rates of mortality and morbidity. Taken together, these data show the critical need to address the initial care of neonatal short bowel syndrome with intestinal failure.

Black children with intestinal failure are at particular risk. Independently of other complications, Black children remained hospitalized for an average of 16 days longer than White children. In considering the Institute of Medicine’s framework for understanding differences vs disparities, increased length of stay in Black children may be due to medical differences or due to social factors, which may be further divided into structural factors and biases.^14^ We did identify medical differences in our cohort between Black and White children, including lower gestational age at birth and more necrotizing enterocolitis diagnoses, suggesting that Black children had more substantial prematurity with a greater number of complications that led to the increased length of stay. However, increased length of stay for Black children was independent of the associations with gestational age and necrotizing enterocolitis diagnosis, suggesting there may be other factors associated with these delays.

Considering social factors that lead to delayed discharge, Black children had lower COI, which may suggest that they were more likely to live in an underresourced area. This may be the enduring result of redlining, a discriminatory banking practice of the 1930s that led to withholding loans predominantly from Black individuals. During the first discharge from the hospital, children with intestinal failure require substantial family support. Family members often must spend time in the hospital demonstrating the ability to care for their children prior to discharge, including learning to manage a central line and parenteral nutrition at home. Families from limited-resource areas may find it more challenging to dedicate that time in the hospital due to income inequities and associated limitations on paid leave, especially in nonsalaried positions. Home nursing can be another critical factor that many caregivers cite as having substantial impact on their abilities to care for their children.^15^ In underresourced areas, home nursing may be more difficult or impossible to obtain, requiring either the family shouldering a greater care burden or the child making further progress before discharge.

Among social factors contributing to disparities, the effects of practitioner bias and structural racism must be considered. Our study adds to a growing body of literature demonstrating that racial differences in outcomes for children in the neonatal intensive care unit cannot be explained by medical differences.^16,17^ In our analysis, adjusting for COI did not eliminate these racial differences. Therefore, we must consider the contributions of factors not measured by medical variables or by COI. This may include practitioner biases or other effects of racism not captured in the COI indicators. Concordance between children and practitioner race, which may mitigate some of these biases, has been demonstrated to reduce disparities in neonatal mortality.^18^ Other considerations may include how racism affects the mother’s care, which may predispose to poor health outcomes in the child.^17^

Differences between those with government or commercial insurance may play an additional role. Those with government insurance remained hospitalized on average 10 days longer than those with commercial insurance. This may again speak to differences in resources available at the time of discharge. Home nursing may be easier to obtain through commercial insurance plans as might other therapies often necessary at initial discharge, such as occupational or physical therapy.

Overall, children with neonatal short bowel syndrome with intestinal failure had prolonged hospitalizations and high costs. The reasons for these outcomes require further study. However, regardless of cause, more rapid discharge to home would benefit hospital systems and patients if done safely.^15^ We propose that standardized discharge criteria and instructions may allow for timelier discharge and may serve to reduce disparities that may be caused by nonclinical factors. Moreover, this may better position clinicians and hospital systems to proactively address insurance-related barriers to discharge through advocacy.

High VTE rates (10%) raise additional concerns. Black children were at greatest risk (14%). As there is lack of uniform screening for this condition, the true incidence may be even higher. In children with intestinal failure, extensive VTE limiting available vascular access sites can be an indication to proceed with intestine transplant. Although the need for intestine transplant has decreased over time, there still remains a substantial portion of children who require the procedure due to limitations in vascular access.^19,20^ The International Intestinal Failure Registry reported a similar VTE incidence in the first year after diagnosis for all children with intestinal failure.^5^ Together, this may shed light on the high risk of VTE early after intestinal failure diagnosis, perhaps during the initial highly inflammatory period with active inflammation from necrotizing enterocolitis or an ischemic volvulus. In conditions such as inflammatory bowel disease, periods of active inflammation correlate with higher thrombosis risk.^21^ Interventions targeting VTE prevention during the initial operative and postoperative period may reduce disease burden.

These results reflect a cohort derived from 50 large freestanding US children’s hospital whose data are represented in PHIS. We believe that this cohort represents the typical child with neonatal-onset intestinal failure that often requires care at a tertiary care children’s hospital. However, this cohort excludes patients not seen at such tertiary care hospitals as well as those at non-US hospitals. Of note, compared with prior multicenter cohorts, this represents one of the broadest samples of intestinal failure patients in terms of number of hospitals represented.^1,4,6^ Thus, we believe this cohort is highly generalizable to the broad pediatric intestinal failure population.

### Limitations

In this observational study, we are limited by existing data available in PHIS and by the use of billing and diagnosis codes. In terms of demographic data, this may have affected race and ethnicity reporting based on medical record data rather than the self-identification of the individual or family. A larger than expected portion of children had unknown race or ethnicity and payer reported. It is unclear whether these children represent a distribution similar to those for whom race, ethnicity, or insurer were reported. Overall, measurement error manifested as misclassification of individuals in the medical record may impact our inferences, although this type of misclassification often results in a type II error rather than a type I error. In each case, missing was a separate category rather than trying to impute values to try to understand what these groups represent. For children with missing gestational age, we used median imputation, which may bias our results. However, excluding gestational age from the analysis or excluding those with missing gestational age had no effect on results. With respect to length of stay, death may be an informative censoring event, yet excluding these patients did not impact the results.

The children identified in this cohort represent those who were receiving parenteral nutrition in the hospital for a minimum of 60 days. However, some patients may be discharged prior to 60 days if they are medically stable to continue home parenteral nutrition. Thus, our cohort may be biased toward individuals requiring longer hospital stays. This may also be affected by comorbidities common in prematurity such as bronchopulmonary dysplasia or intraventricular hemorrhage. Yet, a biased sample with longer hospital stays should make it more challenging to detect differences between groups, suggesting that selection bias should not affect the interpretation of statistical inferences.

## Conclusions

In this cross-sectional study of children with intestinal failure, we developed a novel approach to identify those with neonatal short bowel syndrome with intestinal failure in administrative data that overcomes previous limitations of using diagnosis alone. Black children were overrepresented and had worse outcomes in terms of length of stay, VTE, and CLABSI. Future work should examine individual and societal factors that may lead to these disparities with an eye toward interventions that reduce length of stay and address disparities.

## Notes

Supplement 1. **eTable 1.** Sensitivity analysis of final multivariable length of stay model showing the original model identified by backwards selection, a model using LASSO for variable selection (Model 1), a model excluding those with missing gestational age (Model 2), and a model including only those in the more recent era (Model 3) **eTable 2.** Sensitivity analysis of final multivariable cost model showing the original model identified by backwards selection, a model using LASSO for variable selection (Model 1), a model excluding those with missing gestational age (Model 2), and a model including only those in the more recent era (Model 3) **eTable 3.** Sensitivity analysis of final multivariable survival model showing the original model identified by backwards selection, a model using LASSO for variable selection (Model 1), a model excluding those with missing gestational age (Model 2), and a model including only those in the more recent era (Model 3)

Supplement 2.
Data Sharing Statement
