# Supplementary material for: Social and Financial Costs of Neonatal Intestinal Failure
Source: JAMA Netw Open. 2025 Feb 26;8(2):e2459548. doi: 10.1001/jamanetworkopen.2024.59548 (PMC11866025; doi:10.1001/jamanetworkopen.2024.59548)
Supplement: Supplement 1. — eTable 1. Sensitivity analysis of final multivariable length of stay model showing the original model identified by backwards selection, a model using LASSO for variable selection (Model 1), a model excluding those with missing gestational age (Model 2), and a model including only those in the more recent era (Model 3) eTable 2. Sensitivity analysis of final multivariable cost model showing the original model identified by backwards selection, a model using LASSO for variable selection (Model 1), a model excluding those with missing gestational age (Model 2), and a model including only those in the more recent era (Model 3) eTable 3. Sensitivity analysis of final multivariable survival model showing the original model identified by backwards selection, a model using LASSO for variable selection (Model 1), a model excluding those with missing gestational age (Model 2), and a model including only those in the more recent era (Model 3) [file jamanetwopen-e2459548-s001.pdf]

Supplemental Online Content

Raghu VK, Belaid S, Gutierrez S, et al. Social and financial costs of neonatal intestinal failure. *JAMA Netw Open*. 2025;8(2):e2459548. doi:10.1001/jamanetworkopen.2024.59548

eTable 1. Sensitivity analysis of final multivariable length of stay model showing the original model identified by backwards selection, a model using LASSO for variable selection (Model 1), a model excluding those with missing gestational age (Model 2), and a model including only those in the more recent era (Model 3)

eTable 2. Sensitivity analysis of final multivariable cost model showing the original model identified by backwards selection, a model using LASSO for variable selection (Model 1), a model excluding those with missing gestational age (Model 2), and a model including only those in the more recent era (Model 3)

eTable 3. Sensitivity analysis of final multivariable survival model showing the original model identified by backwards selection, a model using LASSO for variable selection (Model 1), a model excluding those with missing gestational age (Model 2), and a model including only those in the more recent era (Model 3)

This supplemental material has been provided by the authors to give readers additional information about their work.

© 2025 Raghu VK et al. *JAMA Network Open*.

eTable 1. Sensitivity analysis of final multivariable length of stay model showing the original model identified by backwards selection, a model using LASSO for variable selection (Model 1), a model excluding those with missing gestational age (Model 2), and a model including only those in the more recent era (Model 3)

	**Multivariable**		**Model 1**		**Model 2**		**Model 3**	
	Marginal length of stay (95% CI)	p-value	Marginal length of stay (95% CI)	p-value	Marginal length of stay (95% CI)	p-value	Marginal length of stay (95% CI)	p-value
**Female sex**								
**Gestational age in weeks**	-2 (-3 to -2)	<0.001	-3 (-3 to -2)	<0.001	-2 (-3 to -2)	<0.001	-3 (-4 to -2)	<0.001
**Race/Ethnicity (Ref: Non-Hispanic White)**		<0.001		<0.001		<0.001		<0.001
**Hispanic**	+11 (+1 to +20)		+11 (+1 to +20)		+14 (+4 to +24)		+13 (+2 to +23)	
**Non-Hispanic Black**	+16 (+7 to +25)		+18 (+9 to +27)		+19 (+10 to +29)		+21 (+11 to +31)	
**Other***	+7 (-3 to +17)		+7 (-3 to +18)		+9 (-3 to +21)		+10 (-1 to +21)	
**Unknown**	-4 (-15 to +7)		-3 (-14 to +8)		+4 (-6 to +13)		+4 (-11 to +18)	
**Child Opportunity Index (Ref: Very Low)**		0.026		0.027				
**Low**	-2 (-10 to 6)		-3 (-11 to +5)					
**Moderate**	+1 (-7 to 10)		+0 (-9 to +8)					
**High**	+3 (-6 to +12)		+2 (-8 to +11)					
**Very High**	-8 (-18 to +2)		-8 (-18 to +2)					
**Unknown**	+54 (+12 to +96)		+51 (+10 to +92)					
**Payor (Ref: Government)**		<0.001		<0.001				<0.001
**Commercial**	-10 (-17 to -3)		-9 (-16 to -2)				-9 (-17 to -2)	
**Other/Unknown**	+12 (0 to +23)		+14 (+2 to +26)				+53 (+20 to +86)	

© 2025 Raghu VK et al. *JAMA Network Open*.

**Admission after 2009**								
**Diagnosis**								
**Necrotizing Enterocolitis**	+15 (+9 to +21)	<0.001	+16 (+9 to +22)	<0.001	+14 (+7 to +21)	<0.001	+16 (+8 to +23)	<0.001
**Gastroschisis**			+12 (+3 to +22)	0.011				
**Volvulus**			-5 (-18 to +8)	0.448				
**Hirschsprung's Disease**								
**Atresia**								
**CLABSI**	+24 (+14 to +33)	<0.001	+24 (+14 to +33)	<0.001	+26 (+10 to +35)	<0.001	+25 (+14 to +36)	<0.001
**Venous thromboembolism**	+24 (+13 to +34)	<0.001	+24 (+14 to +35)	<0.001	+23 (+10 to +35)	<0.001	+25 (+13 to +37)	<0.001

CLABSI, central line-associated bloodstream infection

*Other includes Asian, Multiracial, and Other categories from the original dataset

© 2025 Raghu VK et al. *JAMA Network Open*.

eTable 2. Sensitivity analysis of final multivariable cost model showing the original model identified by backwards selection, a model using LASSO for variable selection (Model 1), a model excluding those with missing gestational age (Model 2), and a model including only those in the more recent era (Model 3)

	**Multivariable**		**Model 1**		**Model 2**		**Model 3**	
	Marginal cost (95% CI)	p-value	Marginal cost (95% CI)	p-value	Marginal cost (95% CI)	p-value	Marginal cost (95% CI)	p-value
**Length of stay (days)**	+$3791 (+$3172 to +$4410)	<0.001	+$3771 (+$3156 to +$4385)	<0.001	+$4078 (+$3299 to +$4858)	<0.001	+$4408 (+$3488 to +$5329)	<0.001
**Female sex**			-$42,514 (-$74,744 to -$10,284)	0.009				
**Gestational age in weeks**	-$5823 (-$10,083 to -$1563)	0.007	-$6099 (-$10,439 to -$1758)	0.005				
**Race/Ethnicity (Ref: Non-Hispanic White)**				0.16				
**Hispanic**			-$18,193 (-$72,498 to $36,113)					
**Non-Hispanic Black**			-$43,213 (-$93,696 to +$7270)					
**Other***			-$63,456 (-$122,035 to -$4878)					
**Unknown**			-$51,364 (-$118,176 to +$15,448)					
**Child Opportunity Index (Ref: Very Low)**		0.007						
**Low**	+$22,493(-$22,730 to +$67,717)							
**Moderate**	-$28,997 (-$75,508 to +$17,515)							
**High**	+$38,830 (-$12,824 to +$90,484)							
**Very High**	+$82,970 (+$21,181 to +$144,759)							

© 2025 Raghu VK et al. *JAMA Network Open*.

**Unknown**	+$11,411(-$165,943 to +$188,766)							
**Payor (Ref: Government)**		<0.001		0.010		<0.001		
**Commercial**	-$55,069 (-$96,763 to -$13,375)		-$49,211 (-$90,569 to -$7852)		-$28,434 (-$79,445 to +$22,577)			
**Other/Unknown**	-$107,335 (-$160,835 to -$53,835)		-$83,315 (-$151,365 to -$15,266)		-$156,931 (-$227,026 to -$86,835)			
**Admission after 2009**	+$136,798 (+$98,158 to +$194,264)	<0.001	+$143,914 (+$95,861 to +$191,968)	<0.001	+$136,435 (+$76,173 to +$196,696)	<0.001		
**Diagnosis**								
**Necrotizing Enterocolitis**	+$42,168 (+$6721 to +$77,615)	0.019	+$49,581 (+$12,829 to +$86,333)	0.007	+$69,673 (+$27,220 to +$112,125)	0.001	+$57,276 (+$9496 to +$105,056)	0.016
**Gastroschisis**	+78,495 (+$20,915 to +136,074)	0.004	+$69,180 (+$11,718 to +$126,641)	0.014				
**Volvulus**	-$114,275 (-$181,925 to -$46,625)	0.002	-$118,229 (-$185,443 to -$51,015)	0.001	-$142,067 (-$225,509 to -$58,625)	0.001	-$164,380 (-$250,502 to -$78,258)	<0.001
**Hirschsprung's Disease**								
**Atresia**			+$17,274 (-$25,894 to +$60,441)	0.429				
**CLABSI**								
**Venous thromboembolism**								

CLABSI, central line-associated bloodstream infection

*Other includes Asian, Multiracial, and Other categories from the original dataset

© 2025 Raghu VK et al. *JAMA Network Open*.

eTable 3. Sensitivity analysis of final multivariable survival model showing the original model identified by backwards selection, a model using LASSO for variable selection (Model 1), a model excluding those with missing gestational age (Model 2), and a model including only those in the more recent era (Model 3)

	Multivariable		Model 1		Model 2		Model 3	
	Hazard ratio (95% CI)	p-value	Hazard ratio (95% CI)	p-value	Hazard ratio (95% CI)	p-value	Hazard ratio (95% CI)	p-value
**Female sex**							0.60 (0.38 – 0.94)	0.025
**Gestational age in weeks**					0.95 (0.92 – 0.98)	<0.001		
**Race/Ethnicity (Ref: Non-Hispanic White)**		<0.001		0.92				
**Hispanic**	1.30 (0.64 – 2.62)		1.14 (0.55 – 2.37)					
**Non-Hispanic Black**	1.07 (0.53 – 2.15)		1.02 (0.48 – 2.18)					
**Other***	1.12 (0.50 - 2.48)		1.07 (0.49 – 2.36)					
**Unknown**	2.35 (1.30 – 4.27)		1.40 (0.52 – 3.80)					
**Child Opportunity Index (Ref: Very Low)**				0.20				
**Low**			0.95 (0.65 – 1.38)					
**Moderate**			0.69 (0.37 – 1.29)					
**High**			0.51 (0.33 – 0.80)					
**Very High**			0.75 (0.40 – 1.40)					
**Unknown**			0.92 (0.32 – 2.69)					
**Payor (Ref: Government)**				0.17		<0.001		
**Commercial**			1.50 (0.94 – 2.41)		1.27 (0.75 – 2.14)			
**Other/Unknown**			1.49 (0.67 – 3.32)		2.98 (1.73 – 5.13)			
**Admission after 2009**			0.71 (0.43 – 1.16)	0.17				
**Diagnosis**								
**Necrotizing Enterocolitis**								
**Gastroschisis**	0.37 (0.17 – 0.80)	0.011	0.45 (0.19 – 1.09)	0.078			0.27 (0.10 – 0.75)	0.012

© 2025 Raghu VK et al. *JAMA Network Open*.

**Volvulus**								
**Hirschsprung's Disease**								
**Atresia**	0.57 (0.35 – 0.91)	0.020	0.60 (0.37 – 0.97)	0.039				
**CLABSI**	0.58 (0.39 – 0.85)	0.006	0.55 (0.37 – 0.83)	0.005				
**Venous thromboembolism**	1.80 (1.22 – 2.63)	0.003	1.95 (1.30 – 2.94)	0.001	2.02 (1.15 – 3.55)	0.015	2.24 (1.35 – 3.71)	0.002

CLABSI, central line-associated bloodstream infection

*Other includes Asian, Multiracial, and Other categories from the original dataset

© 2025 Raghu VK et al. *JAMA Network Open*.
