# Supplementary material for: Social and Financial Costs of Neonatal Intestinal Failure
Source: JAMA Netw Open. 2025 Feb 26;8(2):e2459548. doi: 10.1001/jamanetworkopen.2024.59548 (PMC11866025; doi:10.1001/jamanetworkopen.2024.59548)
Supplement: Supplement 2. — Data Sharing Statement [file jamanetwopen-e2459548-s002.pdf]

Data Sharing Statement

Raghu. Social and Financial Costs of Neonatal Intestinal Failure. *JAMA Netw Open*. Published February 26, 2025. doi:10.1001/jamanetworkopen.2024.59548

Data
Data available: No

Additional Information
Explanation for why data not available: The data is owned by the Children's Hospital Association. Any requests for data can be made directly to them. Analytic code will be made available by request.
